# Does Local Excision Without Salvage Surgery Affect the Prognostic Outcome of Patients With Malignant Colorectal Polyp? A Long‐Term Survival Analysis

**DOI:** 10.1111/jgh.70506

**Published:** 2026-06-23

**Authors:** Vienna Man Wah Ng, Simon Chu, Anson Huen Yan Chan, Sok Fei Hon, Simon Siu Man Ng

**Affiliations:** ^1^ Department of Surgery Prince of Wales Hospital Hong Kong; ^2^ Department of Surgery The Chinese University of Hong Kong Hong Kong

**Keywords:** endoscopic resection, local excision, malignant polyp, salvage surgery, T1 colorectal cancer

## Abstract

**Background and Aim:**

Management of malignant colorectal polyp has been a treatment dilemma for clinicians. This study aims to evaluate the long‐term survival outcome of patients with and without salvage surgery after local excision.

**Methods:**

From January 2000 to December 2022, patients with T1 malignant colorectal polyp diagnosed after local excision managed by two institutions were included for retrospective review. After propensity score matching, disease‐free, cancer‐specific, and overall survival between local excision alone (LE alone) group and local excision with salvage surgery (LE + SS) group were compared using Kaplan–Meier curves and log‐rank test. Multivariate analysis was performed with Cox regression model to identify independent risk factors affecting disease‐free survival.

**Results:**

Seventy‐nine propensity score–matched pairs were extracted from 309 patients with a mean follow‐up of 65.3 ± 42.6 months. There was no significant difference in the 8‐year disease‐free survival (LE alone, 86.7%; LE + SS, 90.4%; *p* = 0.206), cancer‐specific survival (LE alone, 94.4%; LE + SS, 100%; *p* = 0.114) and overall survival (LE alone, 84.1%; LE + SS, 90.5%; *p* = 0.400). Piecemeal resection (HR, 7.051; 95% CI, 1.856–26.794; *p* = 0.004) and two or more pathological risk factors (HR, 8.552; 95% CI, 1.058–69.095; *p* = 0.044) were independent risk factors for worse disease‐free survival in multivariate analysis. Salvage surgery and presence of single risk factor alone were not associated with disease‐free survival.

**Conclusion:**

Local excision alone is non‐inferior to local excision followed by salvage surgery in terms of long‐term oncological outcome. With better risk profile assessment, local excision alone can be a safe treatment option in patients with T1 colorectal cancer.

## Graphical abstract

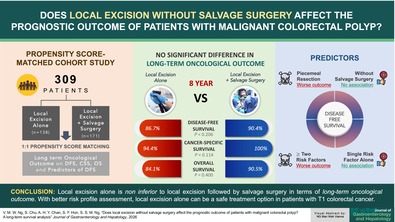

## 1 Introduction

Colorectal cancer was the third commonest cancer worldwide in year 2022 [1] with the highest rate documented in Asia. It is estimated that by year 2040, the burden of colorectal cancer will increase to 3.2 million new cases per year globally (i.e., an increase of 63%) [2]. With the start of colorectal cancer screening program, there has been an increase in the detection of early cancer [3]. The prevalence of T1 malignant polyp diagnosed after endoscopic removal constitutes 40%–60% of stage I disease. Depending on the pathological features of the malignant polyp, the risk of lymph node metastasis ranges from 1.2% to 36.4% and the risk of recurrence ranges from 3.4% to 16.2% [4].

All the existing clinical guidelines [5, 6, 7, 8] recommend additional surgery in patients with any of the following high‐risk histopathological features, such as deep submucosal invasion, poor differentiation, presence of lymphovascular invasion, high grade tumor budding, and positive resection margin. However, > 80% of patients classified as carrying high‐risk features turned out to have no lymph node metastasis after radical colorectal resection [9].

With the advancement of endoscopic and surgical techniques, local excision, such as endoscopic submucosal dissection (ESD) or transanal minimally invasive surgery (TAMIS), has now been regarded as a curative organ‐preserving treatment option for early colorectal cancer. Recent studies suggested that conservative management after local excision could be an oncologically safe alternative to colorectal resection in selected patients [4, 10, 11, 12, 13], with a 5‐year recurrence‐free survival of up to 100% [10]. However, conclusions regarding recurrence and long‐term survival were mixed among the existing large population‐based studies and meta‐analysis [4, 11, 14, 15, 16, 17].

Because colorectal resection is associated with 30% morbidity and 1%–5% mortality [18, 19], it is debatable whether salvage surgery should be performed in all patients with malignant colorectal polyp diagnosed after local excision. It remains challenging for clinicians to select who are suitable for organ preservation strategy and to decide how frequently surveillance should be performed. This study aims to add to the existing literature the long‐term survival outcomes of a sizeable institutional cohort of patients diagnosed with malignant colorectal polyp.

## 2 Methods

### 2.1 Study Population

Patients with T1 malignant colorectal polyp diagnosed after local excision from January 2000 to December 2022 were identified from a prospectively managed database of the New Territories East Cluster hospitals in Hong Kong. Included patients were all managed by the teaching hospital of the Faculty of Medicine of the Chinese University of Hong Kong (Prince of Wales Hospital) and another regional district hospital (North District Hospital). Techniques of local excision are defined as either polypectomy/ endoscopic mucosal resection (EMR), ESD, transanal excision via proctoscope without pneumorectum, or TAMIS. Patients with synchronous colorectal cancer, prior history of colorectal cancer, inflammatory bowel disease or hereditary colorectal cancer syndrome were excluded, as these conditions were associated with higher risk of disease recurrence. Patient demographics, procedural details, endoscopic and histopathological features, as well as follow‐up status were obtained from electronic data records via the Hospital Authority Clinical Management System (HA CMS). This study was approved by the Joint Chinese University of Hong Kong–New Territories East Cluster Clinical Research Ethics Committee (CREC Ref. No. 2023.278). Written informed consent was waived due to the retrospective nature of the study.

### 2.2 Treatment Algorithm

Patients with adenocarcinoma invading into submucosa on pathology report of the local excision specimen would be assessed clinically by the colorectal surgery team of either hospital. Computed tomography (CT) of the thorax, abdomen and pelvis would be performed as systemic staging. Patients with malignant rectal polyp would have additional magnetic resonance imaging (MRI) of the pelvis done as locoregional staging. Serum carcinoembryonic antigen (CEA) level would be taken as baseline tumor marker. In accordance with the European Society of Gastrointestinal Endoscopy (ESGE) and European Society of Digestive Oncology (ESDO) guideline [20], high‐risk T1 colorectal cancer treated locally was defined as the presence of any one of the following histopathological features: (i) poor differentiation, (ii) deep submucosal invasion (i.e., ≥1000 μ m in sessile lesion [21] and Haggitt's level 4 in pedunculated lesion [22]), (iii) lymphovascular invasion, (iv) intense tumor budding, and (v) resection margin ≤ 1 mm or cannot be determined. Patients who were fit for general anesthesia and with high‐risk T1 disease would be offered salvage surgery. Those who had low risk T1 disease, those who refused salvage surgery and those who were unfit for major operation would fall into the group of local excision alone with active surveillance. In instances where pathology reports contained missing or uncertain histopathological details, slide reviews were undertaken and the cases were subsequently discussed in multidisciplinary team meetings to support clinical decision‐making.

### 2.3 Follow‐Up and Surveillance

Patients with and without salvage surgery were followed up regularly by colorectal surgeon, colorectal specialist nurse, and/or clinical oncologist if indicated. Patients with local excision alone would undergo first surveillance sigmoidoscopy or colonoscopy around 6 months after local excision and then at regular intervals. Patients with salvage surgery would have surveillance colonoscopies 1, 3, and 5 years after resection. Interval serum CEA level monitoring would be performed until 5 years after diagnosis. Follow‐up imaging, such as CT of the thorax, abdomen, and pelvis, would be ordered as clinically indicated or at surgeon's discretion.

### 2.4 Outcome Measures

Luminal recurrence was defined as disease recurrence at the previous local excision site or over anastomosis for patients with salvage surgery performed. Locoregional recurrence included luminal recurrence detected endoscopically as well as regional nodal metastasis detected radiologically. Survival time was measured from the date of local excision to the date of the defined event or the date of last follow‐up, whichever occurred first. Disease‐free survival was chosen as the primary outcome of this study, which was defined as the time from local excision to the date at which locoregional recurrence or distant metastasis was first detected. Secondary outcomes included cancer‐specific survival, overall survival, and predictors of disease‐free survival. Cancer‐specific survival was calculated as the time to death from colorectal cancer, while overall survival was calculated as the time to death from any cause.

### 2.5 Statistical Analysis

**FIGURE 1:**
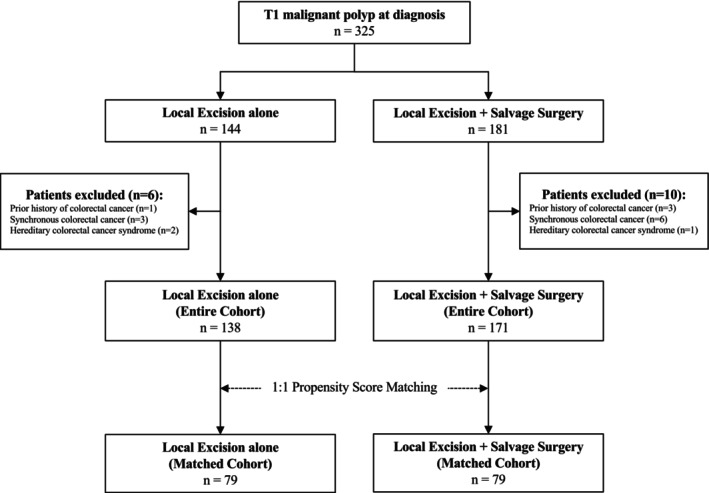
Study flow chart.

Baseline demographics, tumor and treatment characteristics were compared between groups of patients with and without salvage surgery. Continuous variables were compared by *t*‐test while categorical variables were compared by chi‐square test or Fisher's exact test. Because patient and tumor characteristics showed significant differences between the two groups, propensity score (PS) matching was used to adjust for the baseline characteristics of this cohort. The PS was estimated using age, American Society of Anaesthesiologists (ASA) score, tumor differentiation, presence of mucinous component, depth of submucosal invasion, vertical margin, lymphovascular invasion, and the number of pathological risk factors as covariates in multivariable logistic regression analysis. Patients were matched one‐on‐one without replacement using the estimated scores with a caliper at 0.01. After matching, 79 PS matched pairs were extracted from the two groups (Figure 1). Balance diagnostics were achieved with standardized mean differences < 0.25, indicating a good balance and negligible difference between the two groups. Disease‐free survival, cancer‐specific survival, and overall survival were estimated by Kaplan–Meier survival analysis and compared using log rank test. Factors with *p* value of < 0.1 were included in a multivariate Cox regression model for identification of independent risk factors affecting survival. Hazard ratios with 95% confidence intervals were calculated for each parameter. *p* value of < 0.05 was regarded as statistically significant. All tests were two‐sided, and *α* was set at 0.05. SPSS v29.0 was used for all statistical analyses.

## 3 Results

### 3.1 Patient Characteristics

From January 2000 to December 2022, 325 patients with local excision performed for colorectal polyp were diagnosed with T1 colorectal cancer in the two included centers. Sixteen patients with synchronous colorectal cancer, prior history of colorectal cancer, or hereditary colorectal cancer syndrome were excluded. Among the included patients, 171 underwent salvage surgery and 138 had local excision alone. The baseline characteristics of both groups are shown in Table 1. In the entire cohort, there were significant differences observed in age, ASA score, tumor morphology, depth of invasion, vertical margin, and lymphovascular invasion between the local excision alone (LE‐alone) group and the local excision + salvage surgery (LE + SS) group. To adjust for these differences, PS matching was performed. After PS matching, 79 matched pairs of patients were selected. In the matched cohort, the mean age of LE‐alone group and LE + SS group was 65.35 ± 8.71 and 65.58 ± 7.03, respectively. 19.0% patients in LE‐alone group and 20.3% patients in LE + SS group had ASA score 3 or above. There were no longer significant differences between the two groups with respect to tumor location, tumor morphology, tumor differentiation, depth of invasion, vertical margin, and lymphovascular invasion. Regarding pathological risk factors according to the ESGE and ESDO guidelines, 48.1% in LE‐alone group and 50.6% in LE + SS group had two or more high‐risk factors.

**TABLE 1:** Patient and tumor characteristics of entire cohort and propensity score–matched cohort.

	Entire cohort (*n* = 309)			1:1 Matched cohort (*n* = 158)		
	Local excision alone (*n* = 138), *n* (%)	Local excision + salvage surgery (*n* = 171), *n* (%)	*p*	Local excision alone (*n* = 79), *n* (%)	Local excision + salvage surgery (*n* = 79), *n* (%)	*p*
**Age**						
Mean ± SD	67.66 ± 8.67	64.32 ± 7.57	< 0.001*	65.35 ± 8.71	65.58 ± 7.03	0.857
< 65	46 (33.3%)	83 (48.5%)	0.007*	34 (43.0%)	38 (48.1%)	0.523
≥ 65	92 (66.7%)	88 (51.5%)	—	45 (57.0%)	41 (51.9%)	—
**Sex**						
Male	91 (65.9%)	99 (57.9%)	0.148	53 (67.1%)	43 (54.4%)	0.103
Female	47 (34.1%)	72 (42.1%)	—	26 (32.9%)	36 (45.6%)	—
**ASA score**						
ASA 1 or 2	92 (66.7%)	149 (87.1%)	< 0.001*	64 (81.0%)	63 (79.7%)	0.841
ASA 3 or above	46 (33.3%)	22 (12.9%)	—	15 (19.0%)	16 (20.3%)	—
**Local treatment modality**						
Polypectomy/ EMR	117 (84.8%)	123 (71.9%)	0.001*	67 (84.8%)	63 (79.7%)	0.154
ESD	13 (9.4%)	43 (31.0%)	—	7 (8.9%)	14 (17.7%)	—
Transanal excision/TAMIS	8 (5.8%)	5 (2.9%)	—	5 (6.3%)	2 (2.5%)	—
**Piecemeal resection**						
En‐bloc resection	127 (92.0%)	147 (86.0%)	0.094	72 (91.1%)	70 (88.6%)	0.598
Piecemeal resection	11 (8.0%)	24 (14.0%)	—	7 (8.9%)	9 (11.4%)	—
**Tumor location**						
Colon	96 (69.6%)	113 (66.1%)	0.515	48 (60.8%)	58 (73.4%)	0.090
Rectum	42 (30.4%)	58 (33.9%)	—	31 (39.2%)	21 (26.6%)	—
**Tumor morphology**						
Polypoid	96 (69.6%)	76 (44.7%)	< 0.001*	51 (64.6%)	40 (50.6%)	0.077
Sessile	42 (30.4%)	94 (55.3%)	—	28 (35.4%)	39 (49.4%)	—
**Tumor differentiation**						
Well/moderate/NOS	136 (98.6%)	168 (98.2%)	0.833	78 (98.7%)	78 (98.7%)	1.000
Poor	2 (1.4%)	3 (1.8%)	—	1 (1.3%)	1 (1.3%)	—
**Mucinous component**						
No	133 (97.1%)	163 (95.3%)	0.428	77 (97.5%)	78 (98.7%)	0.560
Yes	4 (2.9%)	8 (4.7%)	—	2 (2.5%)	1 (1.3%)	—
**Depth of submucosal invasion**						
Haggitt 1–3 or < 1000 μm	75 (54.3%)	30 (17.5%)	< 0.001*	28 (35.4%)	24 (30.4%)	0.498
Haggitt 4/ ≥ 1000 μm/ uncertain	63 (45.7%)	141 (82.5%)	—	51 (64.6%)	55 (69.6%)	—
**Vertical margin**						
> 1 mm	55 (39.9%)	25 (14.6%)	< 0.001*	20 (25.3%)	22 (27.8%)	0.719
≤ 1 mm/ uncertain	83 (60.1%)	146 (85.4%)	—	59 (74.7%)	57 (72.7%)	—
**Lymphovascular invasion**						
Absent	135 (97.8%)	148 (86.5%)	< 0.001*	76 (96.2%)	76 (96.2%)	1.000
Present	3 (2.2%)	23 (13.5%)	—	3 (3.8%)	3 (3.8%)	—
**Presence of high‐risk features** ^a^						
None	35 (25.4%)	4 (2.3%)	< 0.001*	7 (8.9%)	4 (5.1%)	0.348
Any one or more	103 (74.6%)	167 (97.7%)	—	72 (91.1%)	75 (94.9%)	
**No. of pathological risk factors** ^a^						
≤ 1	91 (65.9%)	40 (23.4%)	< 0.001*	41 (51.9%)	39 (49.4%)	0.750
≥ 2	47 (34.1%)	131 (76.6%)	—	38 (48.1%)	40 (50.6%)	—

Abbreviations: ASA = American Society of Anaesthesiologists, EMR = endoscopic mucosal resection, ESD = endoscopic submucosal dissection, NOS = not otherwise specified, SD = standard deviation, TAMIS = transanal minimally invasive surgery.

* *p* < 0.05 indicates statistically significant.

^a^ Criteria for high‐risk pT1 colorectal cancer that was removed endoscopically according to ESGE and ESDO guideline: (i) poor differentiation, (ii) deep submucosal invasion, (iii) presence of lymphovascular invasion, (iv) intense tumor budding, and (v) uncertain or positive resection margin.

### 3.2 Operative Outcome

Table 2 shows the operative results of patients who had salvage surgery performed in the entire cohort and the matched cohort. In the matched cohort, there was zero surgical mortality and none required reoperation within 30 days. The median hospital stay after operation was 5 (4–8) days. Seventy‐six patients (96.2%) had undergone operation with minimally invasive approach. Nine patients (11.4%) required stoma formation, either temporary or permanent in nature. Two patients (2.5%) had Clavian–Dindo Grade III complications, in which one required image‐guided drainage of intra‐abdominal collection and one required endoscopic hemostasis for anastomotic bleeding. Upon review of the final pathology of the resected specimen, among those with vertical margin ≤1 mm, intraluminal residual cancer was only found in three patients (5.3%) while the remaining 54 patients (94.7%) had no residual luminal malignancy (*p* = 0.556). Regional lymph node metastasis was observed in 12 patients (15.2%) of the matched cohort. Among those with node positive disease, 83.3% patients completed adjuvant treatment.

**TABLE 2:** Operative characteristics of entire cohort and propensity score–matched cohort.

	Entire cohort (*n* = 309)	1:1 matched cohort (*n* = 158)
	Local excision + salvage surgery (*n* = 171), *n* (%)	Local excision + salvage surgery (*n* = 79), *n* (%)
**Surgical approach**		
Minimal invasive surgery (Laparoscopic/ robotic approach)	163 (95.3%)	76 (96.2%)
Open surgery	8 (4.7%)	3 (3.8%)
**Stoma formation (temporary or permanent)**		
No	143 (83.6%)	70 (88.6%)
Yes	28 (16.4%)	9 (11.4%)
**Length of hospital stay (days)**		
Median (IQR)	6 (5–8)	5 (4–8)
**Complication rate**		
No complication/ Clavian–Dindo grade I–II	161 (94.2%)	77 (97.5%)
Clavian–Dindo Grade III or above	10 (5.8%)	2 (2.5%)
Reoperation within 30 days	1 (0.6%)	0 (0.0%)
Mortality	0 (0.0%)	0 (0.0%)
**Luminal residual cancer**		
No	163 (95.3%)	76 (96.2%)
Yes	8 (4.7%)	3 (3.8%)
**No. of lymph nodes harvested**		
Median (IQR)	16 (13–20)	15 (13–20)
**Lymph node metastasis**		
Absent	148 (86.5%)	67 (84.8%)
Present	23 (13.5%)	12 (15.2%)
Completed adjuvant treatment	20 (87.0%)	10 (83.3%)

Abbreviations: IQR = interquartile range.

### 3.3 Disease Recurrence

The median follow‐up period was 64.1 (36.1–94.9) months in the LE + SS group, which was significantly longer than 53.2 (35.4–70.2) months in the LE‐alone group (*p* < 0.001). The loss to follow‐up rate of the entire cohort was 2.59%. Table 3 shows the details of locoregional recurrence and distant metastasis of the entire cohort and the matched cohort. Within the matched cohort, locoregional recurrence was observed in six (7.6%) patients in the LE‐alone group and one (1.3%) patient in the LE + SS group, which showed no significant difference (*p* = 0.117). Distant metastasis was detected in four (5.1%) patients in the LE‐alone group and three (3.8%) patients in the LE + SS group, which also showed no significant difference (*p* = 1.000). Regarding the pattern of disease recurrence, within the LE‐alone group, one patient had isolated luminal recurrence, one patient had isolated pelvic nodal recurrence while one patient had both luminal and regional nodal recurrence; on the other hand, within the LE + SS group, luminal recurrence alone was found in one patient. All these patients were in remission after second surgery. However, three patients from the LE‐alone group developed distant metastasis in addition to locoregional recurrence, requiring second surgery as well as systemic treatment. The clinicopathological details of the eight patients with locoregional recurrence in the entire cohort are summarized in Table 4.

**TABLE 3:** Recurrence and distant metastasis of entire cohort and propensity score–matched cohort.

	Entire cohort (*n* = 309)			1:1 matched cohort (*n* = 158)		
	Local excision alone (*n* = 138), *n* (%)	Local excision + salvage surgery (*n* = 171), *n* (%)	*P*	Local excision alone (*n* = 79), *n* (%)	Local excision + salvage surgery (*n* = 79), *n* (%)	*p*
**Locoregional recurrence**						
No	132 (95.7%)	169 (98.8%)	0.146	73 (92.4%)	78 (98.7%)	0.117
Yes	6 (4.3%)	2 (1.2%)	—	6 (7.6%)	1 (1.3%)	—
Luminal (local excision site/ anastomosis)	5 (3.6%)	1 (0.6%)	—	5 (6.3%)	1 (1.3%)	—
Nodal	5 (3.6%)	1 (0.6%)	—	5 (6.3%)	0 (0.0%)	—
In remission after treatment	6 (100.0%)	2 (100.0%)	—	6 (100.0%)	1 (100.0%)	—
**Metachronous luminal cancer**						
No	138 (100.0%)	170 (99.4%)	1.000	79 (100.0%)	78 (98.7%)	1.000
Yes	0 (0.0%)	1 (0.6%)	—	0 (0.0%)	1 (1.3%)	—
**Distant metastasis**						
No	134 (97.1%)	166 (97.1%)	1.000	75 (94.9%)	76 (96.2%)	1.000
Yes	4 (2.9%)	5 (2.9%)	—	4 (5.1%)	3 (3.8%)	—
Lung	1 (0.7%)	4 (2.3%)	—	1 (1.3%)	2 (2.5%)	—
Liver	3 (2.2%)	1 (0.6%)	—	3 (3.8%)	1 (1.3%)	—
Distant lymph nodes	1 (0.7%)	0 (0.0%)	—	1 (1.3%)	0 (0.0%)	—
In remission after treatment	0 (0.0%)	4 (80.0%)	—	0 (0.0%)	3 (100.0%)	—

**TABLE 4:** Clinicopathological details of the eight cases with locoregional recurrence in the entire cohort.

#	Sex	Age	Tumor location	Pathological risk factors	Primary intervention	Salvage surgery	Type of recurrence	Time to recurrence (months)	Subsequent treatment	Current disease status
1	M	57	Colon	SMI, RM, LVI	Polypectomy	—	LR, NR, DM	58	OT, CT	Alive with metastasis
2	M	62	Rectum	SMI, RM	Polypectomy (piecemeal resection)	—	NR	63	OT	Alive in remission
3	M	62	Colon	SMI, RM	Polypectomy	—	LR	13	OT	Alive in remission
4	F	43	Colon	—	Polypectomy	—	LR, NR, DM	41	OT, CT	Alive with metastasis
5	M	68	Colon	SMI, RM	Polypectomy (piecemeal resection)	—	LR, DM	3	OT	Die with metastasis
6	F	67	Rectum	SMI, RM	Transanal excision (piecemeal resection)	—	LR, NR	5	OT	Alive in remission
7	M	61	Rectum	Mucin, SMI, RM	ESD	TME	NR	51	CT, RT	Alive in remission
8	M	73	Colon	SMI, RM	Polypectomy	Colectomy	LR	62	OT	Alive in remission

Abbreviations: CT = chemotherapy, DM = distant metastasis, ESD = endoscopic submucosal dissection, LR = luminal recurrence, LVI = lymphovascular invasion, NR = regional nodal recurrence, OT = operation, RM = positive or uncertain resection margin, RT = radiotherapy, SMI = deep submucosal invasion, TME = total mesorectal excision.

### 3.4 Survival Analysis

**FIGURE 2:**
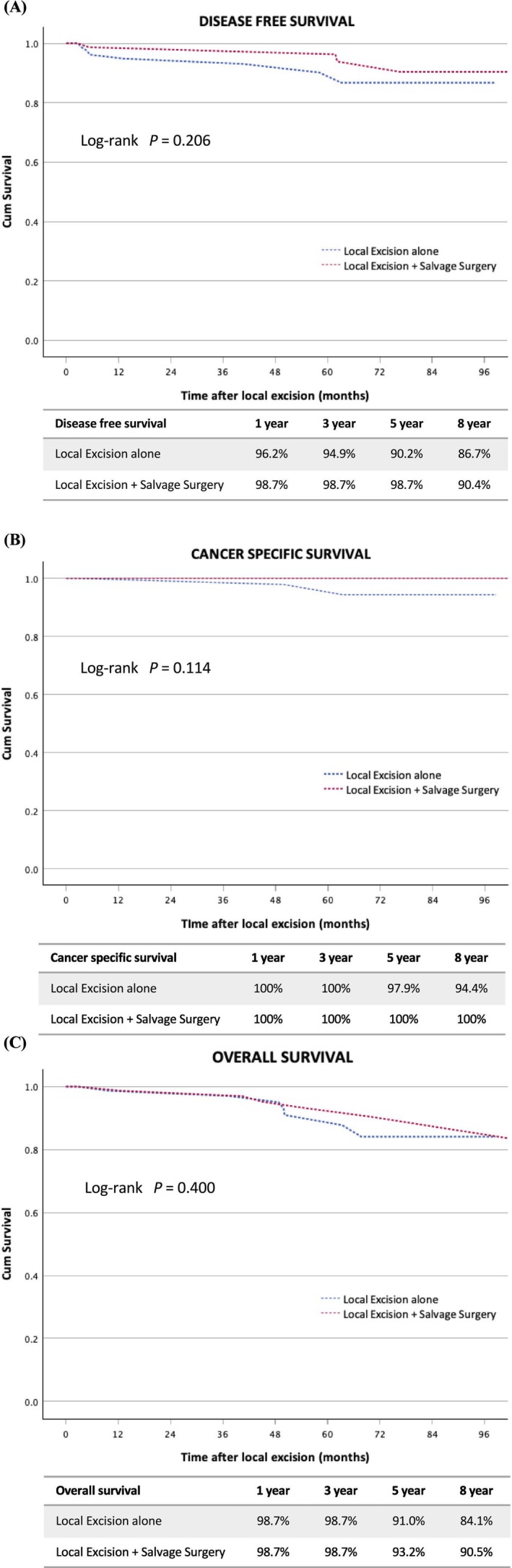
Kaplan–Meier curves for survival rates in the propensity score–matched cohort. (A) Disease‐free survival. (B) Cancer‐specific survival. (C) Overall survival.

The Kaplan–Meier curves of long‐term survival outcomes in the matched cohort are shown in Figure 2. In the LE‐alone group and the LE + SS group, the 8‐year disease‐free survival rates were 86.7% and 90.4%, respectively (*p* = 0.206); the 8‐year cancer‐specific survival rates were 94.4% and 100%, respectively (*p* = 0.114); the 8‐year overall survival rates were 84.1% and 90.5%, respectively (*p* = 0.400). No significant differences were observed in disease‐free survival, cancer‐specific survival, as well as overall survival between the LE‐alone group and the LE + SS group.

### 3.5 Factors Associated With Disease Free Survival

Cox regression analysis was performed to identify risk factors affecting the disease‐free survival of patients with T1 malignant colorectal polyp removed by local excision. Independent risk factors for disease‐free survival with *p* < 0.1 in univariate analysis were piecemeal resection, presence of lymphovascular invasion, and more than one pathological risk factor. Multivariate analysis identified piecemeal resection (HR, 7.051; 95% CI, 1.856–26.794; *p* = 0.004) and two or more pathological risk factors (HR, 8.552; 95% CI, 1.058–69.095; *p* = 0.044) as significant independent risk factors for worse disease‐free survival (Table 5). Salvage surgery and the presence of a single risk factor alone did not show a significant association with disease‐free survival in this study.

**TABLE 5:** Univariate and multivariate analysis of factors affecting disease‐free survival of patients with T1 malignant polyp.

		Univariate analysis		Multivariate analysis	
Factors		Hazard ratio (95% CI)	*p*	Hazard ratio (95% CI)	*p*
Age		0.942 (0.876–1.014)	0.114		
Sex	Male	1.054 (0.308–3.603)	0.933		
	Female				
Tumor location	Colon	0.931 (0.272–3.184)	0.909		
	Rectum				
Tumor morphology	Polypoid	0.475 (0.139–1.627)	0.236		
	Sessile				
Piecemeal resection	Piecemeal resection	5.434 (1.589–18.578)	0.007*	7.051 (1.856–26.794)	0.004*
	En‐bloc resection				
Tumor histology	WD	0.858 (0.251–2.933)	0.807		
	MD/PD/presence of mucin				
Depth of submucosal invasion	Haggitt 4/ ≥ 1000 μ m/ uncertain	4.494 (0.574–35.175)	0.152		
	Haggitt 1–3/ < 1000 μm				
Vertical margin	≤ 1 mm/ uncertain	3.698 (0.473–28.894)	0.212		
	> 1 mm				
Lymphovascular invasion	Present	5.484 (1.180–25.479)	0.030*	5.101 (0.955–27.238)	0.057
	Absent				
Presence of high‐risk features^a^	Any one or more	0.508 (0.064–4.042)	0.522		
	None				
No. of pathological risk factors^a^	≥ 2	9.961 (1.274–77.870)	0.028*	8.552 (1.058–69.095)	0.044*
	≤ 1				
Salvage surgery	No	2.181 (0.633–7.515)	0.217	3.374 (0.928–12.266)	0.065
	Yes				

Abbreviations: 95% CI = 95% confidence interval, MD = moderately differentiated, PD = poorly differentiated, WD = well differentiated.

* *p* < 0.05 indicates statistically significant.

^a^ Criteria for high‐risk pT1 colorectal cancer that was removed endoscopically according to ESGE and ESDO guideline: (i) poor differentiation, (ii) deep submucosal invasion, (iii) presence of lymphovascular invasion, (iv) intense tumor budding, and (v) uncertain or positive resection margin.

## 4 Discussion

The major finding of our present study was that no long‐term differences exist in disease‐free, cancer‐specific, and overall survival in patients with malignant colorectal polyps appropriately triaged to active surveillance compared with salvage surgery. The locoregional recurrence rate of the local excision alone group was 7.6%, which is comparable with the reported rates in surgically treated patients with T1 disease [14]. All patients who developed locoregional recurrence without distant metastasis could be successfully salvaged by subsequent treatment and remained disease free. As reflected by the high 8‐year cancer‐specific survival rate (94.4%) in the local excision alone group, patients did not die from the disease even though recurrence occurred. There was no difference in the distant metastasis rate between the two groups as well. This is by far one of the most encouraging long‐term results available among current literature. Similar retrospective reviews analyzing survival outcome after local resection versus operative management of malignant colorectal polyps are available, but with conflicting results. Levic et al. performed a nationwide retrospective PS‐based analysis from the Danish Colorectal Cancer Group database involving nearly 700 patients [15]. No difference in overall or disease‐free survival was found between the watchful waiting group and the subsequent bowel resection group. This study is particularly important as it first demonstrates the possibility of surgical overtreatment which has not been previously investigated. Our results concur with this study. On the other hand, Lowe et al. carried out another larger retrospective population‐based cohort study from the National Cancer Database run by the American College of Surgeons and the American Cancer Society involving up to 31 000 patients [16]. They demonstrated benefit in overall survival with surgical resection compared with polypectomy alone in patients with malignant colon polyps. However, patients were not matched in this study, and selection bias existed in treatment allocation, especially the polypectomy alone group had a number of factors including higher Charlson Comorbidity Index, non‐insurance status, black race, and rural status that predisposed them to increased overall mortality. Despite large population sample size in this study, information regarding local recurrence and disease‐free survival, which is important to clinical decision, was not available. To specifically investigate the outcome of omitting additional surgery after local excision in high‐risk T1 colorectal cancer, a supplementary PS‐matched analysis from the Japanese Society for Cancer of the Colon and Rectum (JSCCR)‐T1 study was conducted [11]. The finding of this research project was consistent with our present study in demonstrating satisfactory cancer‐specific survival. However, the JSCCR‐T1 study reported a poorer overall survival rate among patients who underwent local excision alone. This discrepancy may be attributed to the absence of non‐oncological variables, such as performance status and comorbidities, in their PS matching. Consequently, the overall survival outcomes could have been influenced by non‐oncological events. Another recent systematic review and meta‐analysis on long‐term outcomes of local resection versus surgical resection for high‐risk T1 colorectal cancer by Chen et al. [4] showed that the net benefit of disease‐specific survival only appeared to be significant when the observation period exceeds 10 years. Therefore, this long‐term net benefit may not be applicable to all patients, especially those with comorbidities. Although this meta‐analysis was the first to compare the long‐term clinical outcomes in patients with high‐risk T1 colorectal polyp, those studies included in this meta‐analysis were mostly retrospective with small case numbers. We believe that findings of our present study could be incorporated as part of the growing evidence in pursuit of identifying appropriate candidates for organ preservation strategy.

To decide on optimal treatment strategy, another important area of concern is the predictors of disease recurrence. Our results indicated that piecemeal resection and presence of two or more pathological risk factors were associated with worse disease‐free survival. A reliable histologic evaluation is dependent on both en‐bloc resection and a thorough pathological examination. Piecemeal resection can result in difficulty in pathological assessment, in particular the depth of invasion and resection margin. With the advancement in diagnostic and therapeutic endoscopy, more accurate endoscopic assessment of the surface and vascular pattern of colorectal lesion is possible via image enhanced endoscopy such as narrow‐band imaging (NBI) [23] and chromoendoscopy with dyes, for example, indigo carmine or crystal violet. Recognition of specific endoscopic features would be essential in choosing the most appropriate method for local excision [24, 25]. When more potential curative options of local excision, for example, ESD, TAMIS, and even endoscopic full‐thickness resection (EFTR), are now available, endoscopists should try every effort to avoid incomplete polypectomy or fragmented polypectomy. If en‐bloc resection is beyond the ability of the endoscopist, patients should be referred to centers with expertise. However, malignant polyp is more commonly diagnosed by pathologists after endoscopic resection without preceding suspicion of invasive malignancy. Previous evidence suggested that high‐risk histopathological features are significantly associated with poor prognosis [5, 6, 7, 8, 26], thus existing international guidelines recommend oncological surgery with adequate regional lymphadenectomy if one or more high‐risk factors are present. However, in our current study, presence of single risk factor is not associated with poor prognosis while presence of two or more risk factors is. Recent studies have also challenged the significance of deep submucosal invasion as sole indication of salvage surgery [14, 27, 28]. Furthermore, pathological analysis of resected specimen in Brisbane series of 239 malignant polyps revealed that the estimated risk of nodal metastasis was only 4.5% when single pathological risk factor was present [29]. In cases with two or more adverse features, the risk of nodal metastasis increased to 23.3%. Therefore, surgical resection should be weighed against patient's comorbidities when only one high‐risk factor is identified. While no single histopathological risk factor could predict whether salvage surgery would offer long‐term survival benefit to patients after local excision, nomogram recently developed to predict the risk of lymph node metastasis may be useful in clinical setting to help selecting patients who may benefit from salvage surgery [30, 31].

The present study has several limitations. First, the study is retrospective in nature. Missing endoscopic and histological features as well as interobserver variability in specimen processing and histopathological assessment introduced potential bias. Although PS matching was applied to reduce selection bias and enhance comparability between the two groups, potential unmeasured confounders may still bias our results. Following matching, only 158 out of the 309 included patients remained available for analysis, with a substantial proportion excluded, thereby reducing the statistical power of the study. Nevertheless, conducting a randomized controlled trial (RCT) in this context is not feasible due to ethical constraints. Furthermore, the rarity of disease recurrence and the long latency to cancer‐related death render adequate statistical power unattainable by RCT. Future multicenter study with larger cohorts, utilizing prospective database and standardized protocol for assessment and surveillance, can be carried out to better evaluate long‐term survival outcomes. Secondly, although current guidelines identify tumor budding as a criterion for high‐risk malignant polyp, we were unable to evaluate its impact on disease‐free survival in this study, as this parameter was reported in only a small number of cases. Standardized pathological assessment and reporting are essential to enable more reliable evaluation of independent prognostic factors for guiding clinical decision. Thirdly, due to limitations of the existing electronic database system, some patients who underwent local excision alone without an electronic operation record prior to the establishment of the registry managed by our colorectal cancer case manager and subsequently defaulted follow‐up in our clinic might have been missed. Nevertheless, we believe the number of such cases is small, as efforts were made to enroll patients who remained under our long‐term survivorship program, even when their local excision had been performed years earlier. Moreover, as the concept of “curative local excision” has only gained clinical attention in our locality over the past decade, the follow‐up period for the local excision alone group was considerably shorter than that of the local excision with salvage surgery group, raising the possibility of detection bias. Accordingly, the survival outcomes in our study should be interpreted with caution. Lastly, as no international surveillance protocol currently exists for patients undergoing local excision without salvage surgery, the surveillance approach—whether interval endoscopy alone or combined with cross‐sectional imaging—varied slightly between the participating institutions. In routine practice, schedules for outpatient follow‐up, CEA monitoring, and surveillance endoscopy were largely standardized across both centers. However, due to resource constraints within the public healthcare system, surveillance imaging such as CT scans could not be performed routinely and was limited to cases with suspected recurrence. This non‐uniform follow‐up strategy may influence the timing of recurrence detection and, consequently, disease‐free survival outcomes. Due to the great heterogeneity, the optimal surveillance interval could not be assessed in current study.

The strength of our study lies in the inclusion of a patient cohort from 2000 to 2022, with a mean follow‐up period exceeding 5 years and a low loss‐to‐follow‐up rate of 2.59%. This study was conducted across two institutions where colorectal surgeons work within the same unit, thereby enabling more consistent patient management and more standardized surgical techniques. Moreover, the surgical outcomes were outstanding, as evidenced by a zero mortality rate and a low complication rate, which collectively contributed to favorable overall survival outcomes. Our study aims to provide long‐term outcomes for patients managed according to their individual clinical and pathological risk profiles in a real‐world setting, rather than to prove that local excision alone or salvage surgery is superior to another in the management of patients with malignant polyp. With the identification of predictors of poorer disease‐free survival, we believe that appropriate treatment selection based on patient and pathological risk profiles can bring about survival benefits.

## 5 Conclusions

In conclusion, local excision alone with active surveillance is non‐inferior to local excision followed by salvage surgery in terms of disease‐free, cancer‐specific and overall survival among patients with malignant colorectal polyps. Long term benefit of salvage surgery exists in selected patients with multiple high‐risk features, but not universally, particularly among those with significant comorbidities and limited life expectancy. With improved risk stratification, local excision alone may represent a safe and personalized treatment option for patients with T1 colorectal cancer.

## Ethics Statement

This study was approved by the Joint Chinese University of Hong Kong—New Territories East Cluster Clinical Research Ethics Committee (The Joint CUHK‐NTEC CREC) in accordance with its standard operating procedure and the principles of the Declaration of Helsinki and ICH Good Clinical Practice. Waiver of Consent was granted for reviewing anonymous patients (CREC Ref. No. 2023.278).

## Conflicts of Interest

The authors declare no conflicts of interest.

## Data Availability

The data that support the findings of this study are available from the corresponding author upon reasonable request.
